# Enhanced cell attachment and hemocompatibility of titanium by nanoscale surface modification through severe plastic integration of magnesium-rich islands and porosification

**DOI:** 10.1038/s41598-017-13169-7

**Published:** 2017-10-11

**Authors:** Masoud Rezaei, Elnaz Tamjid, Ali Dinari

**Affiliations:** 1 Department of Biomaterials, Faculty of High Technologies, Tarbiat Modares University, PO Box, 14115-175 Tehran, Iran; 2 Department of Nanobiotechnology, Faculty of Biological Sciences, Tarbiat Modares University, PO Box, 14115-175 Tehran, Iran

## Abstract

Besides the wide applications of titanium and its alloys for orthopedic and biomedical implants, the biocompatible nature of titanium has emerged various surface modification techniques to enhance its bioactivity and osteointegration with living tissues. In this work, we present a new procedure for nanoscale surface modification of titanium implants by integration of magnesium-rich islands combined with controlled formation of pores and refinement of the surface grain structure. Through severe plastic deformation of the titanium surface with fine magnesium hydride powder, Mg-rich islands with varying sizes ranging from 100 nm to 1000 nm can be integrated inside a thin surface layer (100–500 µm) of the implant. Selective etching of the surface forms a fine structure of surface pores which their average size varies in the range of 200–500 nm depending on the processing condition. I*n vitro* biocompatibility and hemocompatibility assays show that the Mg-rich islands and the induced surface pores significantly enhance cell attachment and biocompatibility without an adverse effect on the cell viability. Therefore, severe plastic integration of Mg-rich islands on titanium surface accompanying with porosification is a new and promising procedure with high potential for nanoscale modification of biomedical implants.

## Introduction

Titanium and its alloys have various applications in biomedical devices mainly in orthopedic and dentistry due to their superior mechanical strength, toughness, biocompatibility, and corrosion resistance^1–5^. Although titanium is chemically active, the presence of the stable oxide layer on its surface makes it naturally passive in physiological environments. Moreover, titanium implants under a harsh environment experience non-specific protein adsorption, interrogation of neutrophils and macrophages, which may lead to encapsulation by fibroblasts^6–8^. In order to improve the biofunctionality of titanium such as bioactivity, osteoconductivity and osteointegration, it is important to modify its surface characteristics^9^. Several techniques have been developed to chemically or physico-chemically modify the surface of titanium implants either through deposition methods or nanostructuring, roughening and porosification^10–14^. Examples are plasma spraying^15^, electrochemical deposition^16^, electrophoretic deposition^17,18^, sol–gel deposition^19^, acid etching^20^, sandblasting^20,21^, physical machining and controlled oxidation^3,22^. While many methods use deposition techniques to cover the surface with a bioactive film, others focus on the surface modification by void formation and nanoscale topography^23^. The presence of surface pores enhance cell attachment, proliferation, differentiation, and bone ingrowth^24^ in expense of reduced stiffness and mechanical strength^25^.

Surface modification of titanium implants by coatings is a general road map to attain enhanced bioactivity. Various synthetic and natural polymers, bioactive glass particles, hydroxyapatite and calcium phosphates as well as their composite structures have been utilized^18,22,26,27^. Recently, surface modification of titanium implants by magnesium and magnesium-based alloys has been found of interest^28–34^. I*n vivo* biological activity of Mg and its key role in osteoblastic cell attachment and bone remodeling processes are very attractive for biomedical applications, particularly for bone repair^28,33,35–40^. The biocompatibility and biodegradability of Mg and its alloys in mammalian cells and tissues have been extensively studied^30,31,36,41–43^. *In vitro* studies have revealed that pure Mg promotes osteoblastic cells proliferation and facilitates ECM protein components expression (such as type I collagen)^33,35,44^. Moreover, *in vivo* investigations on rodents such as rats, guinea pigs and rabbits have indicated that the degradation does not lead to any side effects on the neighboring tissues^42,45,46^. Furthermore, degradation of magnesium in physiological environments releases Mg^+2^ ions that facilitate metabolic reactions and promote the bone formation process^33,36,38,47^. However, there are two key side effects on utilizing Mg/Mg-based alloy implants or coatings including their rapid corrosion in human body  fluid and release of hydrogen gas^28^. The rate of degradation is too fast for the bone tissue to accommodate; hence, the implant loses its mechanical integrity before complete bone healing.

In the present work, we introduce a new procedure to integrate nanoscale magnesium islands inside a surface layer of titanium implants. The lower amount of magnesium in the form of islands, which are integrated in the surface layer, do not adversely affect osteointegration of the implant while providing enhanced bioactivity. The magnesium islands can be leached out either before implantation or degraded in the body, leaving surface pores which further promote cell attachment and osteointegration. We have employed the friction stir processing (FSP) technique and used magnesium hydride to avoid severe magnesium oxidation during severe plastic deformation of the titanium matrix. The solid state nature of this process is essential because the low mutual solubility of titanium and magnesium and their different melting temperature and vapor pressure make processing of such structures impossible. The inherent nature of FSP also refines the surface grain structure of titanium and induces surface roughening which can affect its cellular behavior^48^. This method has recently been utilized for processing of various ultrafine grained materials and composites as well as microstructural modification of castings both in micro- and nano- scales^49^. Limited studies have been carried out on utilizing FSP for biomedical applications, for example, deposition of hydroxyapatite nanoparticles on commercially pure Ti^50^, Mg^51^ and a Mg-Al-Zn alloy (AZ31)^52^. Nevertheless, the process is conceptually of interest for generating bioactive surfaces on titanium implants. We have studied the effect of friction surface integration of Mg islands and formation of surface pores on the microstructure, hardness, biocompatibility and hemocompatibility of commercially pure titanium. The proposed procedure is a new and promising method for nanoscale surface modification of titanium-based implants for biomedical applications.

## Results and Discussion

### Microstructural features after surface plastic deformation

**Figure 1:**
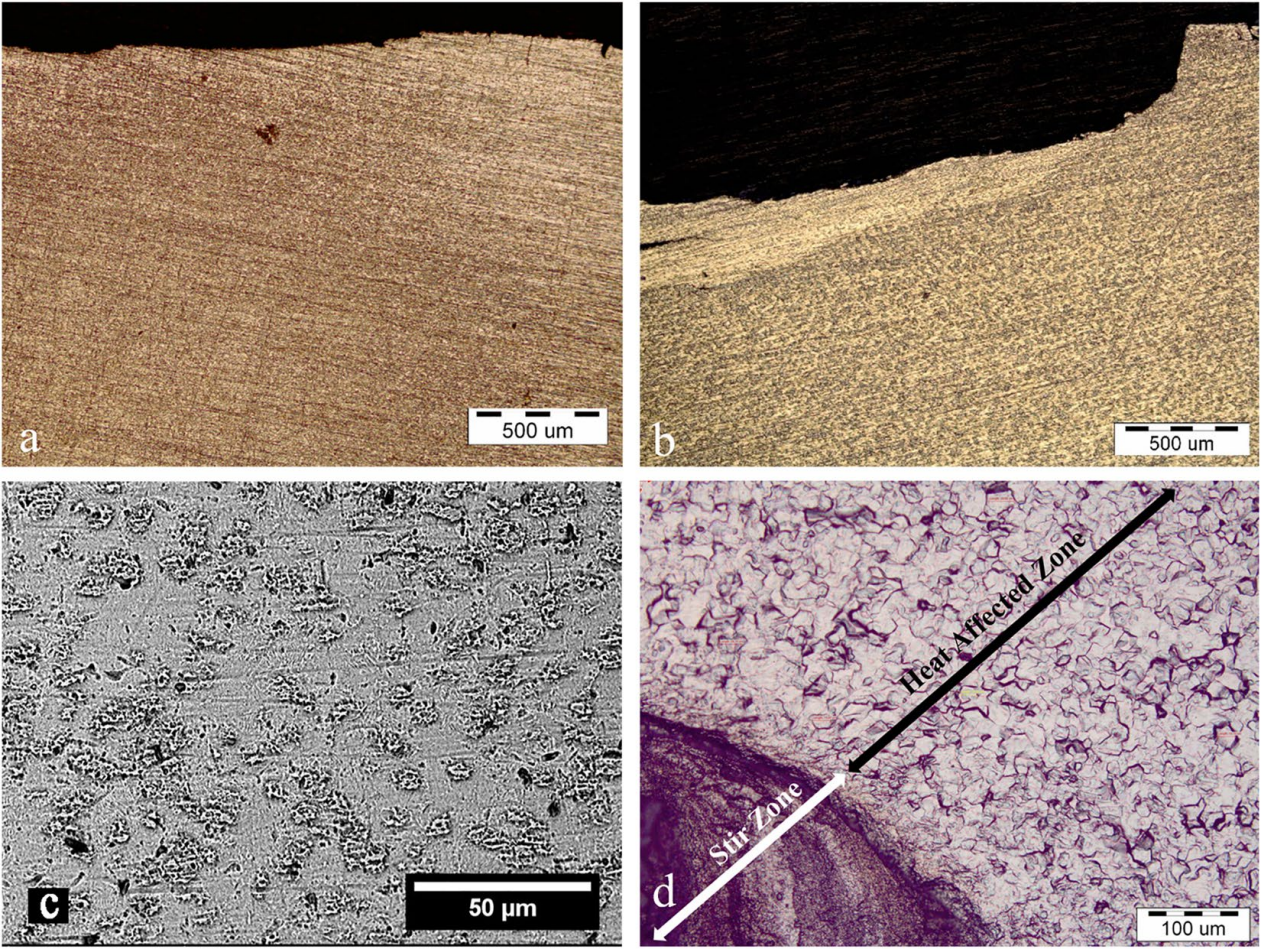
Cross-sectional optical micrographs show the effect of MgH_2_ preplacing and severe plastic integration on the microstructural features of titanium plates. (**a**) Ti surface before treatment. (**b**) Ti surface after plastic deformation at 1250 rpm for 15 s in the presence of MgH_2_ powder. (**c**) Top-view SEM image indicates that the FSI process forms small pores on the surface. (**d**) Optical micrograph shows formation of different zones along the thickness of the titanium plate after FSI.

Figure 1a and b show representative micrographs of the titanium plate before and after pre-placing of magnesium hydride powder and severe plastic integration (FSI). Microstructural examinations determined that the rotating action of the plunge tool stirred and severely deformed the metal matrix, forming a deformed layer (Fig. 1b) with a rough surface (see Fig. S2). Small pores were observed in stir zone (SZ) (Fig. 1c). Small heat affected zone (HAZ) was also developed between the base metal and the severely deformed layer (Fig. 1d). Representative grain structure in SZ and HAZ is shown in ESI S3. The depth of the deformed layer, the average size of grains in HAZ and SZ varied with the processing parameters, as reported in Table 1. It was found that prolonging the rotation time at higher speeds reduced the thickness of the deformed layer, probably because the stirring force pushed the material away from the substrate. A gradient in the thickness of the deformed layer from the middle (maximum thickness) toward the outer region (minimum thickness) was also observed (see ESI S4). This difference depended on the processing condition and could reach to ~120 µm in a severe case.

**Table 1:** Effect of FSI processing parameters on the thickness of the deformed layer and the average grain size of titanium without and with the addition MgH_2_ powder.

Rotational speed (rpm)	800			1250			1600			800	1250
MgH_2_ addition	+			+			+			−	−
Dwell time (s)	15	25	35	15	25	35	15	25	35	15	
Depth of the deformed layer (µm)	470 ± 80	580 ± 90	380 ± 90	320 ± 90	215 ± 90	213 ± 80	110 ± 50	80 ± 40	350 ± 80	*	*
Average grain size in SZ (µm)	5 ± 1.5	3 ± 1.5	3 ± 1.5	4.5 ± 1.5	4 ± 1.5	4 ± 1.5	3.5 ± 1.5	3.5 ± 1.5	3 ± 1	4.5 ± 1.5	4.5 ± 1.5
Average grain size in HAZ (µm)	9 ± 2	9 ± 2.5	9 ± 3	15.5 ± 3	20 ± 3	20 ± 3	20 ± 2	21 ± 4	20 ± 4	4.5 ± 2	11.5 ± 2

**Figure 2:**
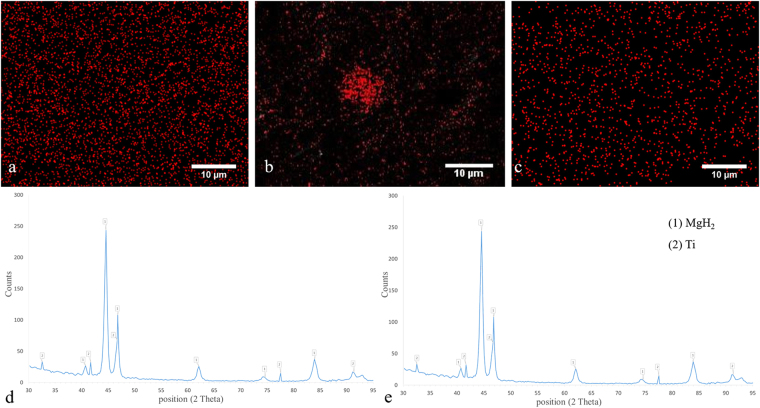
EDS elemental maps show the distribution of Mg (red dots) in the titanium matrix processed at rotational speeds of (**a**) 1250 rpm, (**b**) 800 rpm, and (**c**) 1600 rpm. The dwell time was 15 s. (**d**) XRD pattern indicates the formation of Mg islands inside the titanium matrix after FSI at 1250 rpm for 15 s. (**e**) XRD pattern determines that after chemical etching, Mg was etched out while characteristic peaks of MgH_2_ are still visible.

It is noteworthy that the plastic deformation of the titanium was accompanied by grain refinement induced by dynamic recrystallization mechanisms. Representative microstructural images are shown in S3. The average grain size varied with the processing condition but not with preplacing of MgH_2_ powder (Table 1). The variation of grain size with the processing condition is attributed to the amount of energy input which controls the peak temperature upon severe plastic deformation. As the rotational speed increases and the dwell time decreases, the peak temperature is reduced; hence, the grain refinement becomes limited^53–55^. Nevertheless, many studies have shown that preplacing of hard inclusions promote the grain refinement process during severe plastic deformation due to Zener pinning effect and particle-assisted nucleation mechanisms^56^. During microstructural examinations, it was difficult to detect aggregates, islands or fine dispersions of magnesium or its compounds within the titanium matrix (see Figs 1 and S3). These observations suggested that magnesium hydride particles should be severely refined and probably dissociated and partially dissolved in the titanium matrix due to the severe deformation of titanium by the plunging tool. The formation of supersaturated Mg-Ti alloy during high-energy mechanical milling has recently been shown^34,57^. To support this hypothesis, EDS mapping was performed. Representative results are shown in Fig. 2. The EDS maps indicate fine distribution of Mg within the Ti matrix without formation of large Mg-based agglomerates. Notably, the size of Mg-containing regions was significantly smaller than the initial magnesium hydride powder. Interestingly, the Mg distribution became more homogeneous when the rotational speed was decreased and/or the dwell time was prolonged. Chemical analysis indicated that more magnesium was integrated within the titanium matrix at higher rotational speeds (see Table S1), which could be due to immediate groove coverage at the beginning of the process. Meanwhile, the characteristic XRD peaks of α-MgH_2_ were detected (Fig. 2e), which revealed that particle refinement and distribution in the titanium matrix occurred.

### Surface porosification

**Figure 3:**
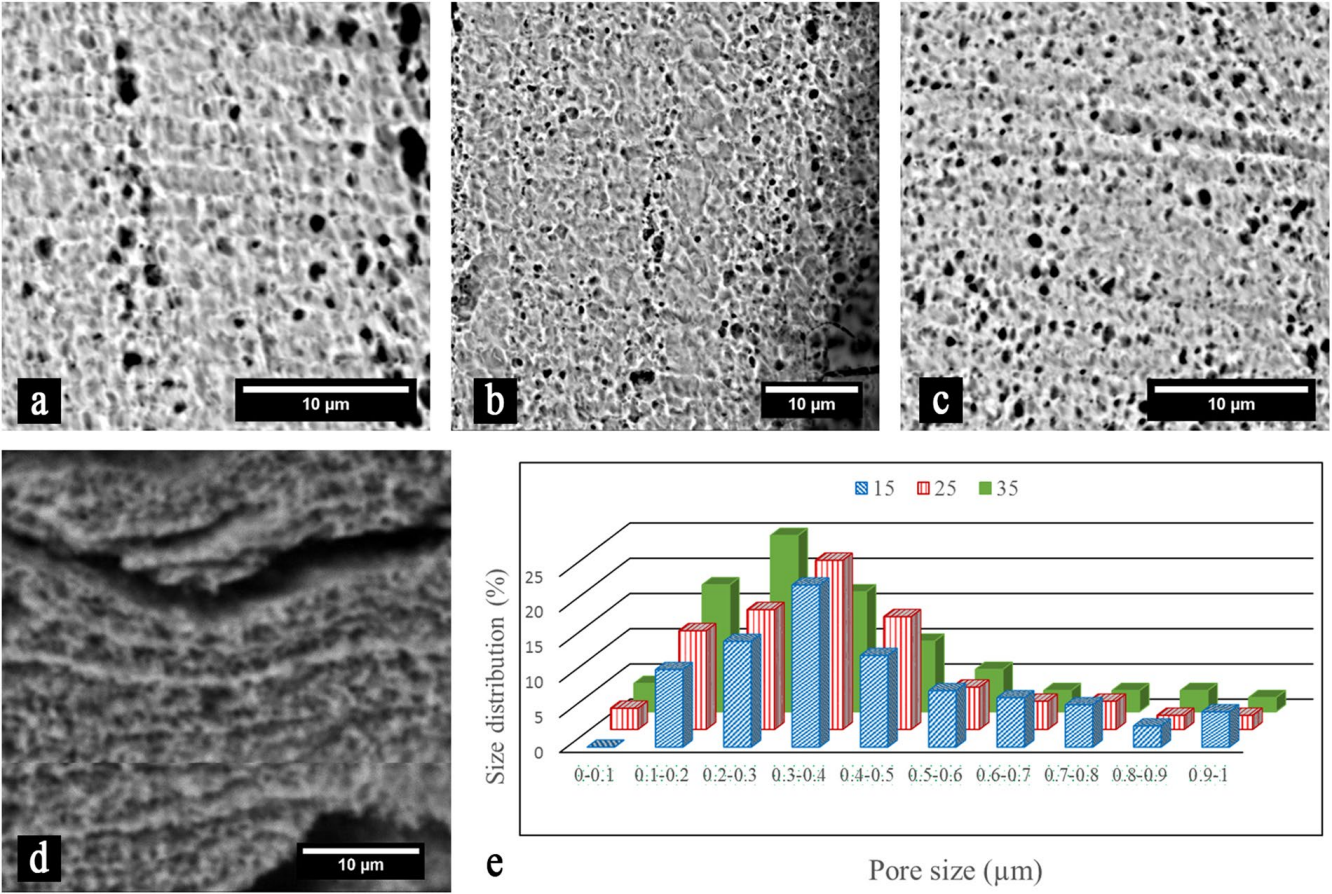
Representative SEM images show porosification of the FSI-treated titanium plate after chemical etching for 30 s. The specimens were prepared at rotational speed of 1250 rpm with a dwell time of (**a**) 15 s, (**b**) 25 s, and (**c**) 35 s. (**d**) Pore coalescence forms large voids and cracks. (**e**) Histogram shows the distribution of pore sizes dependent on the dwell time (s)

In order to form nanopores on the surface of titanium implants, the Mg-rich islands were leached out by short chemical etching. Representative SEM images of SZ after 30 s etching is shown in Fig. 3. The results reveal that the Mg-rich islands are chemically active with the Karol solution, leading to a fast corrosion rate and formation of fine pores. The sizes of the induced pores mostly vary between 200 to 500 nm depending on the processing conditions (Fig. 3a–c). Generally, smaller pores are formed at higher rotational speeds and prolonged times (higher energy inputs). In some conditions and locations, coalescence of the pores to form larger voids and cracks is noticed (Fig. 3d). Although pores are formed even at a short immersing time in the corrosive solution (15 s), pore opening becomes after 30 s. It is also noticeable that the morphology of the formed pores in the Mg-rich islands is very different than the base metal which is pitting type. Figure 3e demonstrates the effect of parameters on pore size distribution and indicates that the mean size is reduced with increasing the dwell time and the rotational speed. It is pertinent to note that the majority of the pores are in the range of 200–500 nm. In order to study the residual of Mg-islands on the surface after chemical etching, EDX elemental mapping and XRD were performed. It was found that the concentration of magnesium significantly reduced on the surface of the titanium implant after chemical etching (see Fig. S3 and Table S1). Nevertheless, XRD pattern (Fig. 3d) revealed that MgH_2_ islands still existed in the deformed layer. This observation suggested that the decomposed magnesium hydride was leached out and leaved pores, while a small portion of the untransformed particles remained in the matrix due to a higher corrosion resistance.

### Surface hardness

**Figure 4:**
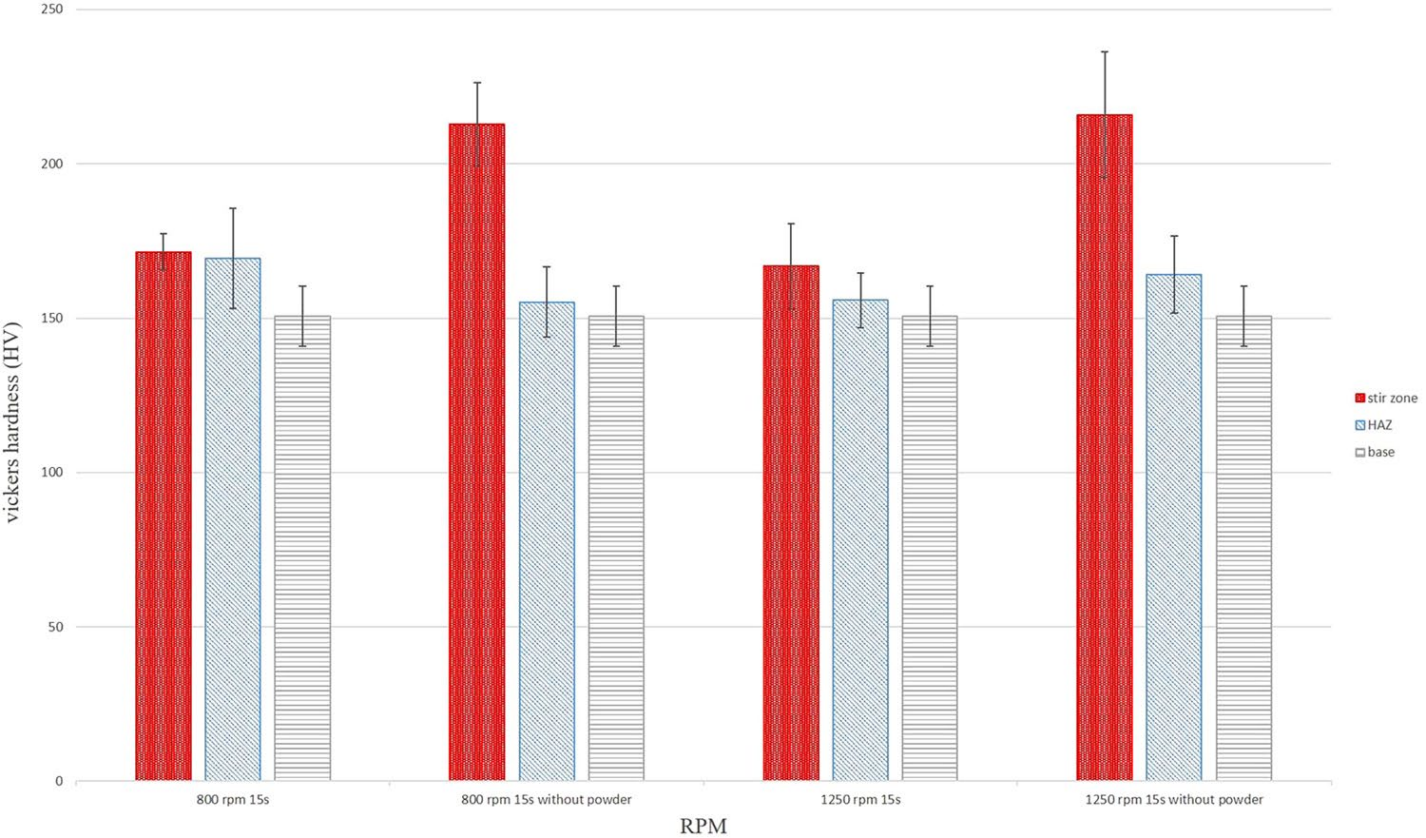
Effect of surface treatment on the hardness of different zones for FSI-treated (1 and 3) Ti + MgH_2_ and (2 and 4) Ti. The rotational speed and dwell time was (1 and 2) 800 rpm and 15 s and (3 and 4) 1250 rpm and 15 s.

Mechanical surface treatment of titanium plates by the rotating tool plastically deforms the metal matrix and refines its grain structure. Therefore, surface hardening is very susceptible. Figure 4 shows the effect of surface treatment on the micro-hardness values of HAZ and SZ for Ti and Ti + MgH_2_ specimens. The surface hardness of the as-received titanium increased from ~158 HV to ~216 HV after mechanical treatment. Previous microstructural study on friction stir welding of commercially pure titanium has indicated that recrystallized grains of SZ contain a large number of twins and a high density of dislocations^55^. Limited slip systems in titanium activates twinning mechanisms for the deformation while the high density of dislocations highlights incomplete recovery process^54^. Anyway, the enhanced surface hardness is attributed to severe plastic deformation and the refined grain structure. Notably, the hardness of the specimens containing magnesium hydride particles is lower than pure titanium. As shown in Table 1 and Fig. 3, the grain structure is finer for pure titanium while islands of decomposed MgH_2_ are presented. Therefore, lower surface hardness was attained.

### Cell studies

**Figure 5:**
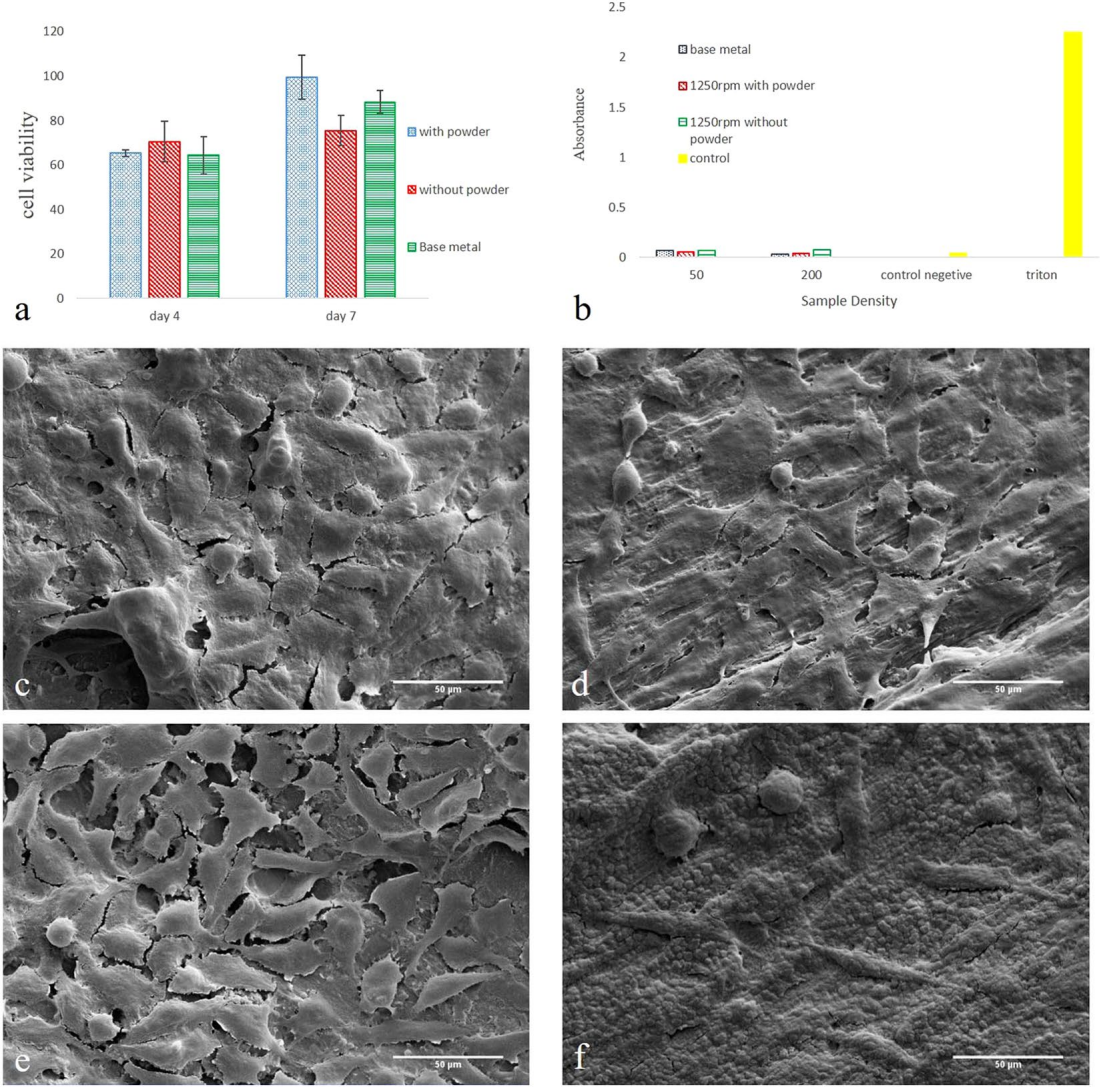
Results of cell studies showing the effect of mechanical surface treatment and Mg-rich islands on the biocompatibility, cell attachment and hemocompatibility of examined titanium implants. (**a**) MTT assay incubation for 7 days indicates that cell viability is enhanced by the magnesium treatment. (**b**) Hemolysis reveals that the Mg-rich islands slightly improved blood compatibility of titanium. SEM images show L929 cell attchemt on the surface of FSI-treated titanium after 7 days of incubation. The processing condition was: (**c**) 1250 rpm for 15 s in the presence of MgH_2_ and after 80 s chemical etching; (**d**) 1250 rpm for 15 s and after 80 s chemical etching; (**e**) 1250 rpm for 15 s in the presence of MgH_2_; (**f**) 1250 rpm for 15 s.

The results of biocompatibility studies on the surface modified samples in L929 and human RBC lines are shown in Fig. 5. Untreated (as-received) titanium implant was used as control. The specimen processed with magnesium hydride possess higher cell viability in MTT assay after 7 days of incubation as compared with the control and the processed specimen without magnesium (Fig. 5a). No adverse effect of the Mg-rich islands on the hemocompatibility was also noticed (Fig. 5b). Representative SEM images of the samples incubated with L929 cells for 7 days are shown in Fig. 5c to f. The cell density on the surface of the samples containing

Mg-rich islands is higher than the control. From the morphological point of view, the cells are significantly spread on the surface, while the presence of multiple filopodia extended from the cell to the substrate provides an evidence of the cell attachment to the surfaces. After porosification on both titanium plates without and with Mg-rich islands, multiple layers of cells are observed. This observation reveals the positive influence of nanopores on the cell attachment. The results also indicates that cell attachment is improved (higher cell density) when Mg-islands are integrated in the surface as compared with treated Ti without MgH_2_.

## Materials and Methods

### Severe surface integration of magnesium islands

A commercially pure titanium sheet ASTM G2 (>99.2wt. % purity) with a thickness of 3 mm was supplied from TIMET (Gerenzano, Italy). The amount of carbon and iron impurity was 0.1 and 0.3 wt.%. The average grain size of the titanium matrix was 9 ± 2 μm (see Electronic Supplementary Information (ESI) S1). The sheet was cut into small plates with dimensions of 60mm × 100mm by a mechanical cutter. A groove with dimensions of 1.5 mm (wide) and 2 mm (depth) was machined in the middle of the plates and filled with a commercial magnesium hydride powder (98wt. % purity; Alfa Aesar, USA). The powder had an average particle size of 50 μm with a narrow size distribution (see ESI S1). A cylindrical tungsten carbide tool with a shoulder diameter of 20 mm, probe diameter of 10 mm, and probe length of 1.5 mm were used to disperse the magnesium hydride powder inside the titanium substrate. The tool angel and plunge depth were 0° and 2 mm. Rotational speeds of 800, 1250 and 1600 rpm for dwell times of 15, 25 and 35 s were employed. The process was performed in spot mode under an argon atmosphere to prevent oxidation of the active metals. To create surface porosity, some of the plates were immersed in Karol solution (3 ml HF and 4 ml HNO_3_ in 53 ml distilled water) for different times (from 15 to 80 s) to study the effect of etching time on the pore size. For comparison, titanium plates without pre-placing MgH_2_ powder were also processed with the same condition.

### Materials characterizations

Microstructural studies were performed by optical microscopy (DP25, OLYMPUSE, Japan) and scanning electron microscopy (SEM, ProX, Phenome, Nederland). The distribution of magnesium in the titanium matrix and the effect of etching on the amount of magnesium before and after etching were analyzed by SEM (VEGA, TESCAN, Czech Republic) equipped with energy-dispersive X-ray spectroscopy (EDS). Metallographic specimens were prepared by standard techniques using SiC emery papers, alumina suspensions (for mechanical polishing), and Karol chemical etching. Phase analysis was carried out by X-ray diffraction (XRD, XʹPert MPD, PHILIPS, Netherland). Cross-sectional micro-hardness measurements were done by Vickers method (60044, Buehler LTD, USA). The applied load was 1000 grf and the dwell time was 15 s.

### Biocompatibility assay

To examine the effect of surface modification on the cell viability, cubes with dimensions of 3 × 3 × 3 mm^3^ were machined from stir zone of the plates. Prior to cell seeding, the samples were cleaned and sterilized by sonication in acetone (Merck, Germany) and 70% ethanol aqueous solution (HAMONTEB, Iran) for 15 min, and then UV treatment for 30 min. *In vitro* biocompatibility of the plates was studied by 3-(4, 5-dimethylthiazol-2-yl)-2, 5-diphenyl tetrazolium bromide (MTT) assay (Sigma-Aldrich, USA) using mouse fibroblast connective tissue cell lines (L929, Iran Biological Resource Center) as a gold standard cell line in cytotoxicity assays. The MTT assay was performed based on standard protocols. Briefly, the cells were cultured in Dulbecco’s modifier medium (DMEM, sigma- Aldrich, USA) with 10% Fetal Bovine Serum (FBS, Sigma- Aldrich, USA) at 37 °C in humidifier atmosphere with 5% CO_2_. The cells with a surface density of 1 × 10^4^ cells/cm^2^ were seeded on the samples and incubated up to 7 days at 37 °C in 5% CO_2_. At different time intervals, 5 mg/ml of the MTT solution was added to each well and the cells were incubated for 4 h. Afterwards, the formed formazan crystals were solubilized within dimethyl sulfoxide (DAEJUNG, South Korea) for 15 min and the absorbance at 570 nm was measured for each plate by ELISA reader (BioTek microplate reader, USA).

The morphology and density of the incubated cells attached to the surface of the specimens were examined by SEM. For this aim, cells with a density of 2 × 10^4^ were seeded on the 12-well plates containing samples. After 7 days incubation, the cells were fixed with 4 vol% paraformaldehyde for 20 min. Finally, the samples were gold coated and observed under a low-vacuum digital SEM (KYKY-EM3200, Switzerland).

### Hemocompatibility

Blood compatibility assay was performed according to the *hemolysis assay* standard procedure^58^. Human blood was obtained from normal donors by vein puncture. The blood was collected in EDTA and centrifuged at 13000 rpm for 2 min to separate red blood cells (RBC) from blood plasma of donors. The viability of the cells was always >99.5% as estimated by trypan blue dye exclusion. The cells were washed with phosphate buffer saline (PBS) for 3 times, and followed by supernatant removal and centrifugation at 1300 rpm for 5 min. This process was repeated twice, and finally a dilute suspension of RBC in PBS (108 Uni/ml) with a pH of 7.4 was prepared.

To access the blood compatibility, the surface modified specimens (~10 g) were incubated in 3 ml PBS at 37 °C for 72 h. The PBS was extracted and mixed with the RBC suspension (1:4 volume ratio) in a tube and centrifuged at 13000 rpm for 5 min. The absorbance was measured at 540 nm by the ELISA reader. 1% Triton X-100 was used as positive control, and the buffer alone was used as negative control.

### Statistical analysis

Assays were performed 3 times and the results were presented as mean ± SD. One-way ANOVA test was performed with a statistical significance of *p* < 0.05.

### Compliance of ethical standards

Human blood was obtained from normal donors by vein puncture, while the consent was obtained from all of participants as written. Medical ethics committee of Tarbiat Modares University (Tehran, Iran) approved this study.

## Conclusions

In this work, a new procedure was introduced to integrate magnesium-rich islands in the surface layer of titanium implants. Due to the low solubility of Mg in Ti matrix, a solid-state surface deformation technique was employed to locally incorporate magnesium islands in few hundred micrometers (100–500 µm) of the surface layer through mechanical stirring and plunging of MgH_2_ particles. It was shown that MgH_2_ particles were partially decomposed and distributed inside the titanium matrix. The distribution of the islands could be controlled by the processing conditions including the rotational speed of the tool and dwell time. SEM studies coupled with EDS mapping revealed that the size of Mg-rich islands varied in the range of 100 to 500 nm. Using short chemical etching, small pores were formed on the surface layer. The average size of the pores mostly varied between 200 to 500 nm depending on the processing condition. Smaller pores were attained at higher energy inputs, i.e. increased/prolonged rotational speed/time. The refined grained structure along with severe plastic deformation of titanium significantly hardened the surface layer. Lower hardness values were measured in the presence of MgH_2_, revealing the role of magnesium islands on the dynamic recrystallization and grain refinement process. *In vitro* biocompatibility assay showed improved cell viability for the Ti plates containing Mg-rich islands. Hemolysis did not show adverse effect of the surface nanomodification on the human blood compatibility. An improved cell adhesion was also observed for the treated samples, particularly after porosification. Therefore, friction stir integration of Mg-rich islands in the surface of titanium is potentially a promising approach for the surface modification of the implants for biomedical applications.

## Electronic supplementary material

Electronic Supporting Information
