# Supplementary material for: Enhanced cell attachment and hemocompatibility of titanium by nanoscale surface modification through severe plastic integration of magnesium-rich islands and porosification
Source: Sci Rep. 2017 Oct 11;7:12965. doi: 10.1038/s41598-017-13169-7 (PMC5636805; doi:10.1038/s41598-017-13169-7)
Supplement: Supplementary file 1 — Electronic Supporting Information [file 41598_2017_13169_MOESM1_ESM.doc]

*Electronic Supporting Information*

**Enhanced cell attachment and hemocompatibility of titanium by nanoscale surface modification through severe plastic integration of magnesium-rich islands and porosification**

Masoud Rezaei ^1^, Elnaz Tamjid ^2^*, Ali Dinari ^2^

^1^Department of Biomaterials, Faculty of High Technologies, Tarbiat Modares University, PO Box 14115-175, Tehran, Iran

^2^Department of Nanobiotechnology, Faculty of Biological Sciences, Tarbiat Modares University, PO Box 14115-175, Tehran, Iran

*Corresponding author: Tel: +98 (21) 8288 4746; Fax: +98 (21) 8288 4717, E-mail: [tamjid@modares.ac.ir](mailto:tamjid@modares.ac.ir)

[Masoud.rezaei92@yahoo.com](mailto:Masoud.rezaei92@yahoo.com) / masoud.rezaeeii@modares.ac.ir

**
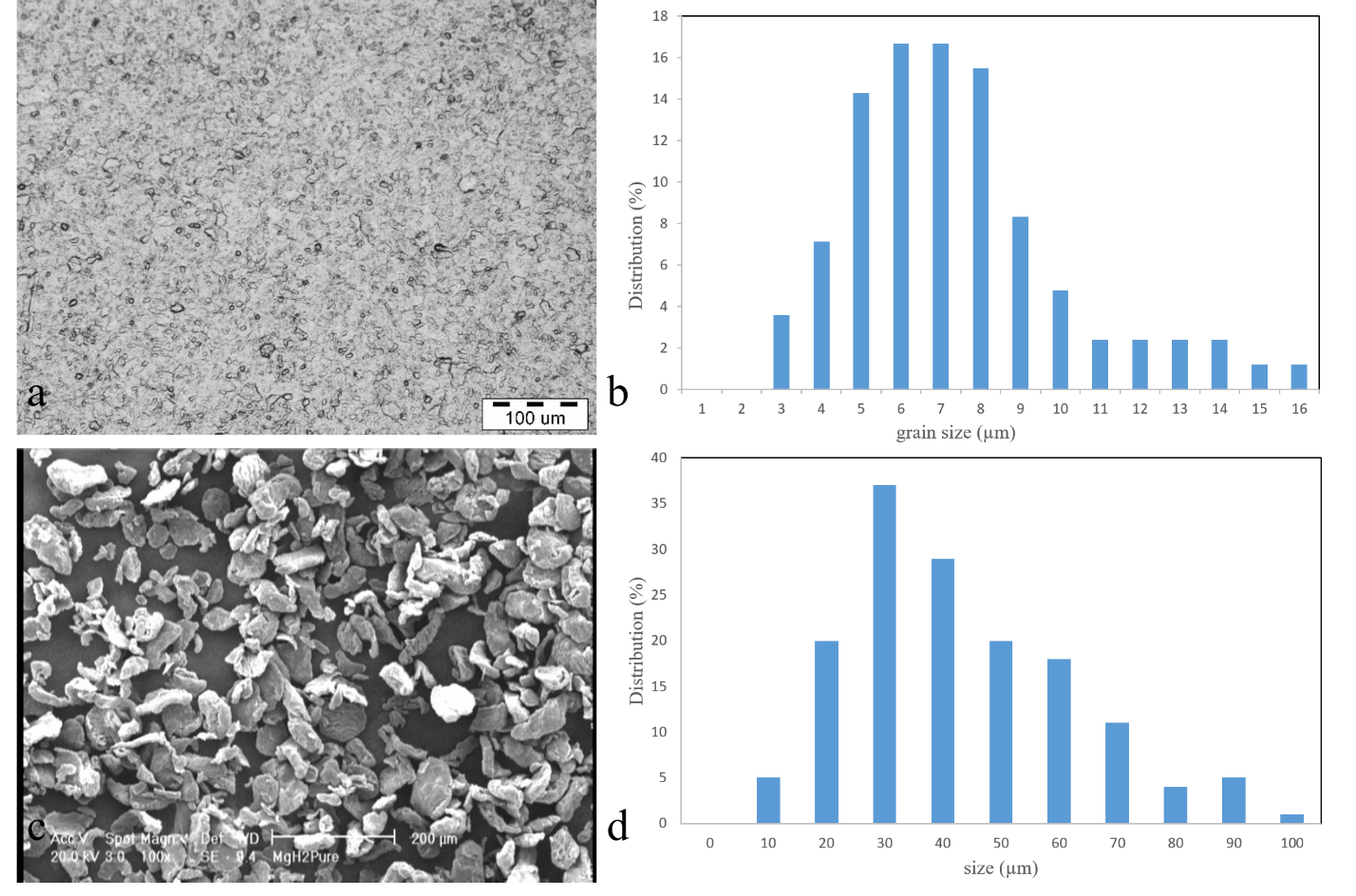
**

**Fig S1:** **(a)** Optical micrograph shows the grain structure of titanium sheet. **(b)** Histogram shows the size distribution of titanium grains. **(c)** SEM micrograph shows the size and morphology of MgH_2_ particles. (d) Size distribution of MgH_2_ powder.


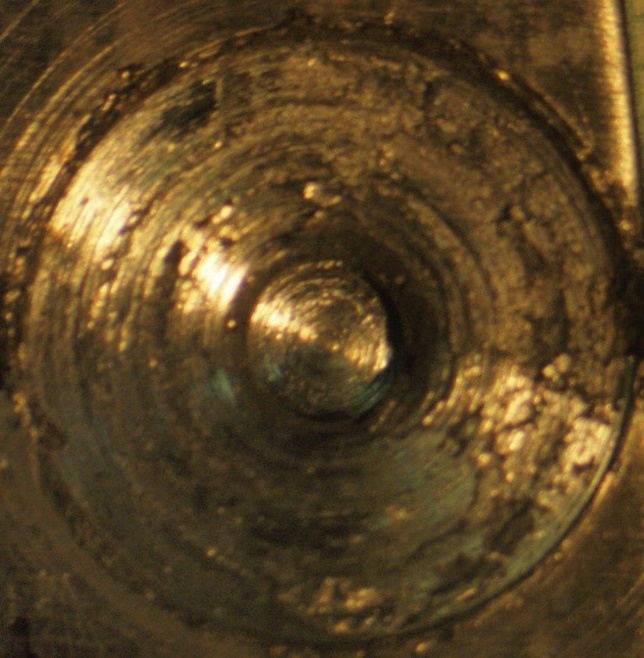


**Fig. S2:** Optical micrographs reveal that the surface of titanium plate becomes rough after FSI.

**
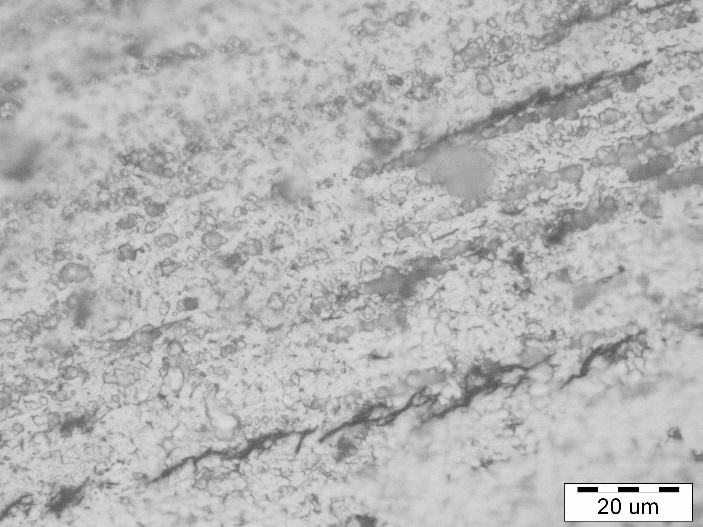
**
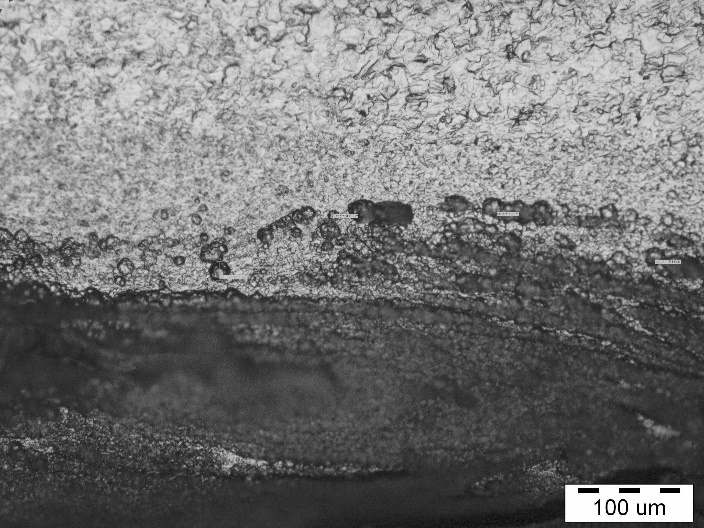


a

b

**
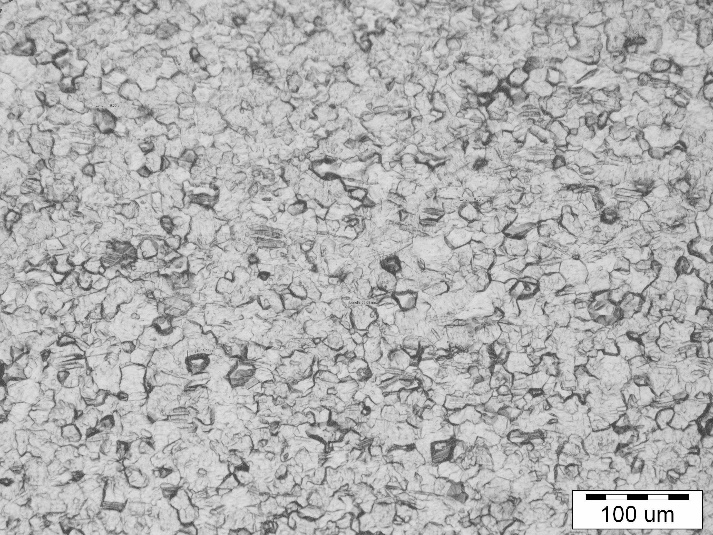
**
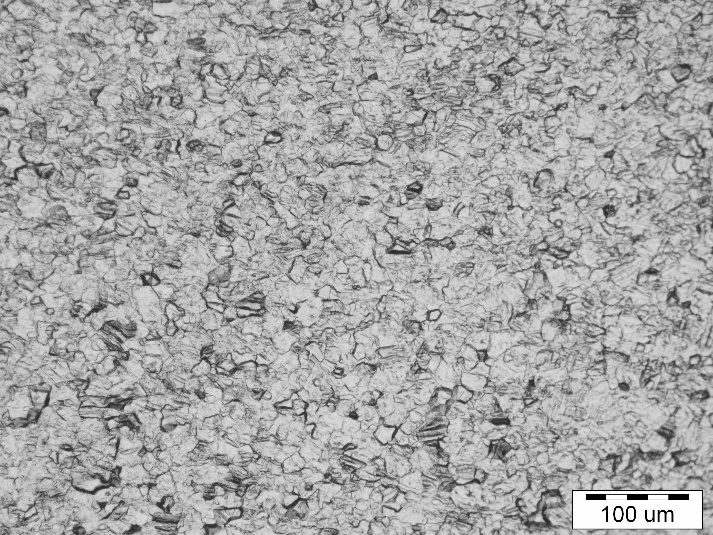


c

d

**Fig. S3:** Representative optical micrographs show the grain structure of FSI-treated Ti plate in **(a, b)** SZ and **(c, d)** HAZ. The rotational speed was **(a, c)** 1250 and **(b, d)** 1600 rpm. The dwell time was 15 s.


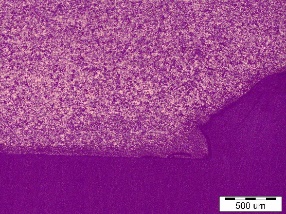

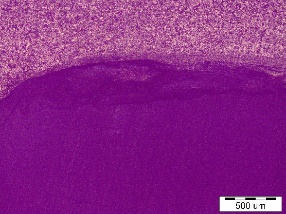

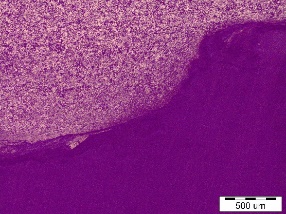

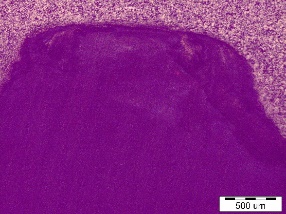

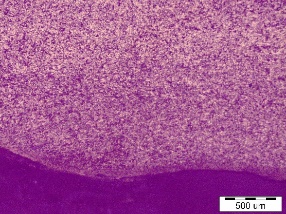

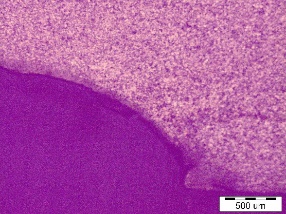


**Fig. S4:** Bonded optical images indicates variation in the thickness of the deformed layer after FSI.

**Table S1:** EDS analysis shows variations of the magnesium concentration (wt%) in the FSI-treated Ti plates processed at different rotational speeds before and after chemical etching

| Rotational speed (rpm) | 800 | 1250 | 1600 | 800 | 1250 | 1600 |
| --- | --- | --- | --- | --- | --- | --- |
| Chemical etching | - | - | - | + | + | + |
| Ti | 89.6 | 98.5 | 98.3 | - | 99.7 | - |
| Mg | 0.6 | 1.4 | 1.5 | - | 0.2 | - |
| O_2_ | 9.8 | 0.1 | 0.2 | - | 0.1 | - |


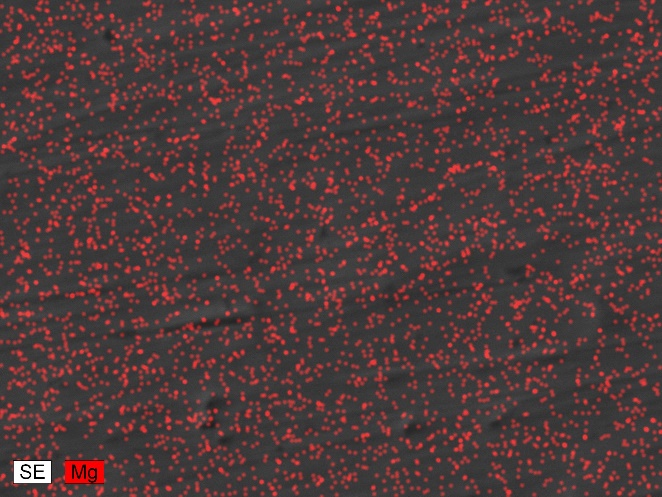

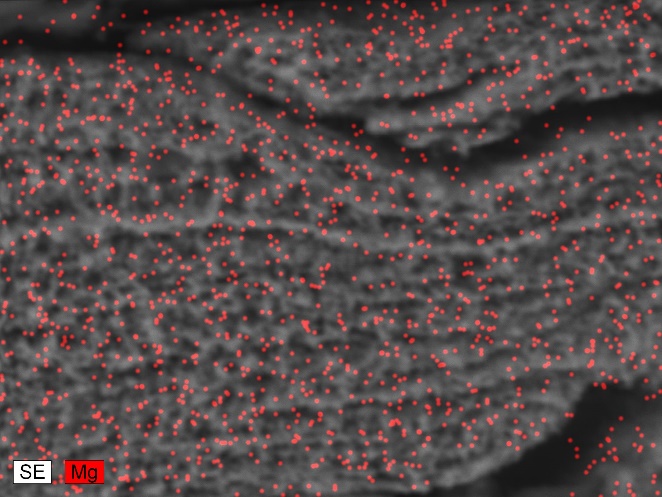


**Fig. S3:** EDX elemental mapping indicates removal of Mg-rich after short chemical etching. **(a)** The titanium plate was FSIed at 1250 rpm for 15 s. **(b)** The treated plate was chemically etched for 80 s.
